# Analysis of the Degradation of Broad-Spectrum Cephalosporins by OXA-48-Producing *Enterobacteriaceae* Using MALDI-TOF MS

**DOI:** 10.3390/microorganisms7120614

**Published:** 2019-11-26

**Authors:** Marina Oviaño, María Rosario Rodicio, Jürgen J. Heinisch, Rosaura Rodicio, Germán Bou, Javier Fernández

**Affiliations:** 1 Servicio de Microbiología, Complejo Hospitalario Universitario A Coruña, 15006 La Coruña, Spain; Marina.Oviano.Garcia@sergas.es (M.O.); German.Bou.Arevalo@sergas.es (G.B.); 2 Instituto de Investigación Biomédica A Coruña, 15006 La Coruña, Spain; 3 Department of Functional Biology, Section of Microbiology, University of Oviedo, 33011 Oviedo, Spain; rrodicio@uniovi.es; 4 Instituto de Investigación Sanitaria del Principado de Asturias, 33011 Oviedo, Spain; 5 Fachbereich Biologie/Chemie, Universität Osnabrück, 49069 Osnabrück, Germany; Juergen.Heinisch@biologie.uni-osnabrueck.de; 6 Department of Biochemistry and Molecular Biology, University of Oviedo, 33006 Oviedo, Spain; mrosaura@uniovi.es; 7 Servicio de Microbiología, Hospital Universitario Central de Asturias, 33011 Oviedo, Spain

**Keywords:** OXA-48, MALDI-TOF, broad-spectrum cephalosporins

## Abstract

The objective of the study was to evaluate the activity of OXA-48 against different broad-spectrum cephalosporins and to identify the reaction products by MALDI-TOF MS. The action of OXA-48 on cefotaxime, ceftazidime, and ceftriaxone was assessed by this method, using an *Escherichia coli* J53 transconjugant carrying only the ~62 Kb IncL plasmid containing the *bla*_OXA-48_ gene, and the same strain without any plasmid was included as a negative control. In addition, a collection of 17 clinical OXA-48-producing *Enterobacteriaceae*, which were susceptible to broad-spectrum cephalosporins, was evaluated. MALDI-TOF MS-based analysis of the *E. coli* transconjugant carrying the *bla*_OXA-48_-harboring plasmid, and also the clinical isolates, showed degradation of cefotaxime into two inactive compounds—decarboxylated and deacetylated cefotaxime (~370 Da) and deacetyl cefotaxime (~414 Da), both with the hydrolyzed beta-lactam ring. Reaction products were not obtained when the experiment was performed with ceftriaxone or ceftazidime. From a clinical point of view, our study supports the idea that the efficacy of cefotaxime against OXA-48-producing *Enterobacteriaceae* is doubtful, in contrast to ceftazidime and ceftriaxone which could be valid choices for treating infections caused by these bacteria. However, further clinical studies confirming this hypothesis are required.

## 1. Introduction

The oxacillinases comprise a heterogeneous group of class D β-lactamases that are able to hydrolyze amino- and carboxypenicillins and, for some members of the group, also cephalosporins and carbapenems to a greater or lesser extent [1,2]. OXA-48 is a carbapenem-hydrolyzing OXA-type enzyme which was first identified in a carbapenem-resistant *Klebsiella pneumoniae* recovered from a patient in Turkey in 2004 and then spread worldwide to different species of *Enterobacteriaceae* [2,3,4]. Currently, OXA-48 is the most frequently detected carbapenemase among carbapenem-resistant-*Enterobacteriaceae* in many countries of the Middle East, North Africa, and Europe [5,6]. Therapeutic options against infections caused by carbapenemase-producing *Enterobacteriaceae* are scarce and consequently these bacteria represent a major threat for patient safety worldwide [6]. OXA-48 and its variants, with the exception of OXA-163, hydrolyze carbapenems at low level, but do not confer resistance to broad-spectrum cephalosporins in contrast to other carbapenemases whose activity against these drugs is well known [2,4]. Nonetheless, previous studies showed that the catalytic activity (Kcat/Km) of OXA-48 for cefotaxime is moderate, whereas it is practically null for ceftazidime or cefepime [3,7]. Consequently, from the point of view of clinical efficacy, in vivo experiments in mice evidenced that therapy with ceftazidime was effective against OXA-48-producing *Enterobacteriaceae* lacking extended-spectrum beta-lactamases (ESBLs) or AmpC-like β-lactamases, whereas the therapy with cefotaxime had little impact in reducing lethality of the rodents [8,9].

Since at least some cephalosporins may be a possible alternative for the treatment of invasive infections caused by OXA-48-producing *Enterobacteriaceae*, the aim of the present study was to evaluate the activity of OXA-48 against three commonly used broad-spectrum cephalosporins, for which data are still limited (cefotaxime, ceftazidime) or unavailable (ceftriaxone), and to analyze the reaction products, using matrix-assisted laser desorption/ionization time-of-flight mass spectrometry (MALDI-TOF MS).

## 2. Materials and Methods

A previously characterized OXA-48-producing *K. pneumoniae* strain (Kp-HUCA-4; [10]) carrying the *bla*_OXA-48_ gene without any other resistant determinant in the original ~62-Kb IncL/M plasmid (later reclassified as IncL) was chosen for a preliminary experiment. A conjugation assay was performed using Kp-HUCA-48 as donor and the sodium azide-resistant *Escherichia coli* J53 as the recipient strain. Transconjugants were selected on eosin methylene blue agar containing sodium azide (100 mg/L) plus ertapenem (0.5 mg/L), and plasmid DNA was extracted using the Kado and Liu technique [10]. The activity of OXA-48 against broad-spectrum cephalosporins was assessed by an MALDI-TOF MS assay, using the J53 transconjugant carrying only the ~62 Kb IncL plasmid containing the *bla*_OXA-48_ gene. The J53 strain lacking the plasmid was included as a negative control. Isolates were previously grown overnight at 37 °C in trypticase soy agar with 5% sheep blood (Becton Dickinson, Heidelberg, Germany). The activity on cefotaxime (Sigma-Aldrich, Hamburg, Germany; 0.50 mg/mL), ceftazidime (Sigma-Aldrich; 0.25 mg/mL), and ceftriaxone (Sigma-Aldrich; 0.25 mg/mL) was evaluated after resuspension of the bacteria that filled a 1 μL inoculation loop in 50 μL of the antibiotic solution in buffer containing 10 mM NH_4_HCO_3_, 10 µg/mL ZnCl_2_, and 0.001% SDS, as previously described [11]. The suspension was incubated at 37 °C under agitation during 30 min for ceftriaxone, 60 min for cefotaxime, and 3 h for ceftazidime as previously described for the MALDI-TOF MS analysis of ESBL enzymes [11]. The tubes were then centrifuged for 2 min at 13,000× *g* at room temperature, and 1 μL of the supernatant was applied mixed with 1 μL of α-cyano-4-hydroxycinnamic acid (IVD Matrix HCCA-portioned, Bruker Daltonik, Bruker Daltonics GmbH, Bremen, Germany). Spectra were acquired in an MALDI Microflex LT/SH bench-top mass spectrometer (Bruker Daltonik), equipped with a 60 Hz nitrogen laser in the mass range between 100 and 1000 Da, with a mass peak resolution >300 by employing an optimized acquisition method using FlexControl v.3.4 software (Bruker Daltonics GmbH). MALDI-TOF MS analysis, calibration, and spectra processing were carried out as previously reported [11,12]. The MBT STAR-BL Software module (Bruker Daltonics GmbH) was used to evaluate the spectra. The STAR-BL module was used for automated interpretation of spectra, by calculating the logRQ value which indicates the rate of degradation for the different antibiotics by calculating the logarithmic ratio of the intensity of the mass peaks of the hydrolyzed antibiotic in the intensities of the non-hydrolyzed antibiotic. LogRQ values were normalized according to defined negative and positive control strains. Specifically, *E. coli* ATCC 25922 was used as negative control and a PCR-confirmed VIM-1-producing *E. coli* was the positive control. Normalized logRQ values below 0.2 represent negative results, while values above 0.4 indicate beta-lactamase activity. Normalized logRQ values between 0.2 and 0.4 correspond to an ambiguous result [12,13]. Next, the same protocol was applied to a collection of 17 clinical OXA-48-producing *K. pneumoniae* (8) and *E. coli* (9) isolates that did not coproduce extended-spectrum beta-lactamases (ESBL), AmpC beta-lactamases, or other carbapenemases, all of them susceptible to cefotaxime (MIC ≤ 1).

## 3. Results and Discussion

**Figure 1:**
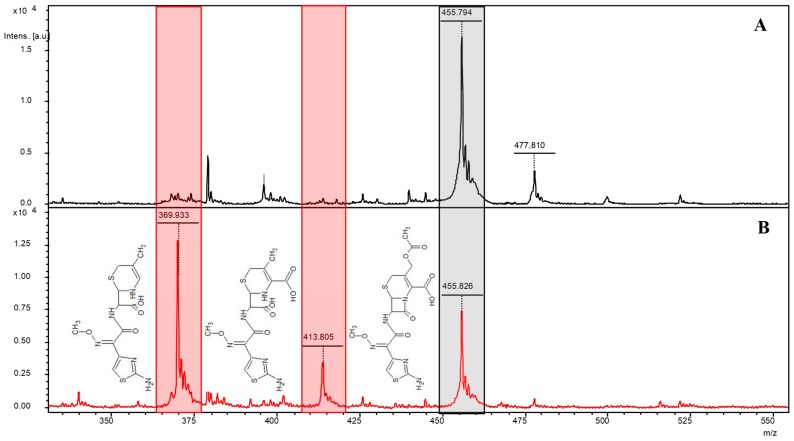
Mass spectra of cefotaxime exposed to lysates of *Escherichia coli* strains producing and not producing OXA-48. (**A**) Mass spectra of cefotaxime 60 min after exposure to the bacterial lysate of the *E. coli* J53 control strain. (**B**) Mass spectra of cefotaxime 60 min after exposure to the bacterial lysate of the *E. coli* J53 transconjugant carrying the ~62-Kb IncL plasmid with the *bla*_OXA-48_ gene. In both images, the black shading corresponds to the mass peak of cefotaxime (~455 Da), whereas the red shading corresponds to the mass peaks of the two degradation products, namely decarboxyl and deacetyl cefotaxime (~370 Da) and deacetyl cefotaxime (~414 Da), both with the hydrolyzed beta-lactam ring. The chemical structure of the compounds is shown next to each peak.

MALDI-TOF MS-based analysis of the transconjugant carrying the bla_OXA-48_-harboring plasmid showed degradation of cefotaxime into two products of ~414 and ~370 Da. However, these products did not appear in the experiment performed with the same strain lacking the plasmid, used as negative control (Figure 1). Furthermore, no reaction products were obtained when the experiment was performed with ceftriaxone or ceftazidime (not shown). Mass peaks of ~414 and ~370 Da have previously been reported as a consequence of cefotaxime degradation by ESBL-producing *Enterobacteriaceae* [11,13]. The ~370 Da peak corresponds to a decarboxylated and deacetylated derivative of cefotaxime in the hydrolyzed form, while the ~414 Da peak is deacetyl cefotaxime also hydrolyzed [14].

Both compounds are microbiologically inactive. However, the fact that hydrolysis of cefotaxime is not complete can explain why OXA-48-producing enterobacterial isolates remain phenotypically susceptible to cefotaxime in vitro. Activity on cefotaxime can also explain the eventual positive results obtained by colorimetric assays based on pH reduction, aimed at ESBL detection [15,16], which could be due to carboxyl-acid formation after hydrolysis of the beta-lactam ring. From a clinical point of view, the efficacy of cefotaxime against OXA-48-producing *Enterobacteriaceae* could be impaired by the aforementioned hydrolysis and this is consistent with the previously reported lower efficacy of this drug in vivo with respect to ceftazidime or cefepime [8,9].

**Figure 2:**
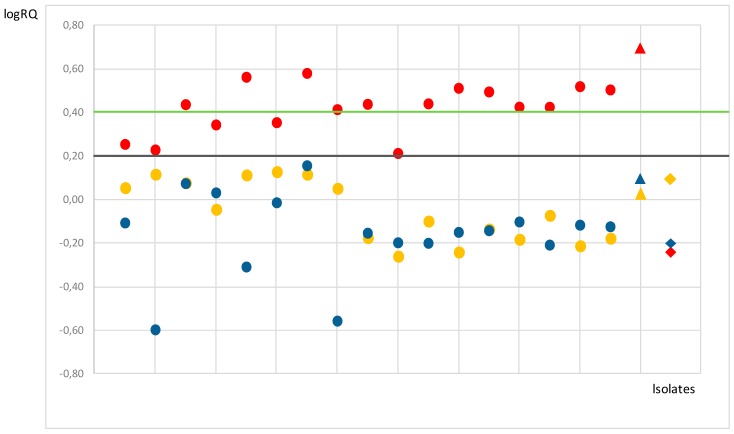
Comparison of normalized cefotaxime (red), ceftriaxone (yellow), and ceftazidime (blue) logRQ values for 17 OXA-48 producers (clinical isolates of *Klebsiella pneumoniae* and *Escherichia coli*) represented by dots. LogRQ values above the cut-off are considered positive (0.4, green line) and values below the cut-off (0.2, black line) are considered negative. Intermediate values (between the green and black lines) are ambiguous. The *E. coli* J53 control strain and the transconjugant of the latter carrying the ~62-Kb IncL plasmid with the *bla*_OXA-48_ gene are represented by diamonds and triangles, respectively, using the same color code as in the experiment.

When the MALDI-TOF MS-based experiment was carried out with 17 OXA-48-producing *K. pneumoniae* and *E. coli* clinical isolates, similar results were observed. As shown in Figure 2, the logRQ cut-off value established by the STAR-BL module demonstrated a clear degradation of cefotaxime (logRQ > 0.4) by the transconjugant carrying the bla_OXA-48_-harboring plasmid and most of the OXA-48-producing clinical strains (12/17). The remaining isolates (5/17; 2 of *K. pneumoniae* and 3 of *E. coli*) showed ambiguous patterns (logRQ between 2 and 4) for cefotaxime. Consistent with the aforementioned results, activity against ceftazidime and ceftriaxone (logRQ < 2) was not evidenced for any of the tested strains (not shown).

The activity of different ESBLs against broad-spectrum cephalosporins has been previously assessed by MALDI-TOF MS [11,12,13,14]. However, as far as we know, this is the first study in which hydrolysis of these drugs by the OXA-48 carbapenemase has been evaluated by this approach. Moreover, the action of OXA-48 on ceftriaxone, a drug widely used in hospitals to fight different infections, has been here investigated for the first time. According to our results, ceftriaxone, like ceftazidime, could be a valid choice for the treatment of infections caused by OXA-48-producing *Enterobacteriaceae* that do not coproduce ESBLs or AmpC-like β-lactamases. Although some studies in animal models have been published for ceftazidime and cefepime [8,9], further in vivo studies and clinical experience are required for all broad-spectrum cephalosporins. Unfortunately, cefepime degradation could not be evaluated due to the poor specificity of the MALDI-TOF MS-based assay previously reported for this drug [12].

## 4. Conclusions

The present study confirmed the degradation of cefotaxime by the OXA-48 enzyme, providing further evidence to rule out the use of this antibiotic for the treatment of OXA-48-producing *Enterobacteriaceae*. Our results also support the conclusion that ceftazidime and ceftriaxone could be valid choices for treating infections caused by these bacteria, if they do not have other mechanisms conferring resistance to broad-spectrum cephalosporins. The use of these drugs could be of great interest for hospital antimicrobial stewardship initiatives, leading to a reduction in the usage of carbapenems against OXA-48-producing *Enterobacteriaceae* infections.
